# Exploring the Intersections of Trauma, Structural Adversity, and Psychosis among a Primarily African-American Sample: A Mixed-Methods Analysis

**DOI:** 10.3389/fpsyt.2017.00057

**Published:** 2017-04-19

**Authors:** Cherise Rosen, Nev Jones, Eleanor Longden, Kayla A. Chase, Mona Shattell, Jennifer K. Melbourne, Sarah K. Keedy, Rajiv P. Sharma

**Affiliations:** ^1^Department of Psychiatry, University of Illinois at Chicago, Chicago, IL, USA; ^2^Felton Institute, San Francisco, CA, USA; ^3^Greater Manchester West Mental Health NHS Foundation Trust, Psychosis Research Unit, Manchester, UK; ^4^Department of Psychiatry, University of California San Diego, La Jolla, CA, USA; ^5^Department of Community, Systems, and Mental Health Nursing, Rush University, Chicago, IL, USA; ^6^Department of Psychiatry, University of Chicago, Chicago, IL, USA; ^7^Department of Psychiatry, Jesse Brown Veterans Affairs Medical Center, Chicago, IL, USA

**Keywords:** psychosis, traumatic life events, delusions, hallucinations, mixed methods

## Abstract

Traumatic life events (TLEs) have been associated with multiple psychiatric diagnoses, including anxiety disorders, major depression, PTSD, and psychosis. To advance our understanding of the complex interactions between forms of adversity as they manifest across the lifespan, psychosis, and symptom content, we undertook a mixed-methods investigation of TLEs and psychosis. Our research explored the association between cumulative exposures, type of TLE, and proximity to the traumatic event and psychosis; the association between TLEs and clinical symptomology including specific types of delusions and/or hallucinations; and how qualitative data further inform understanding of complex relationships and patterns of past trauma and symptoms as they unfold over time. There were a total of 97 participants in the quantitative study sample, 51 participants with present state psychosis and 46 non-clinical. There were a total of 34 qualitative study participants, all of whom were experiencing psychosis. The quantitative analysis showed that when comparing persons with psychosis to the non-clinical group, there were no group differences in the overall total score of TLEs. However, there was a significant difference in cumulative TLEs that “Happened,” demonstrating that as the number of TLEs increased, the likelihood of clinical psychosis also increased. We also found a correlation between lifetime cumulative TLEs that “Happened” and PANSS five-factor analysis: positive, excitement, depression, thought disorder, activation, and paranoia scores. The qualitative analysis further built on these finding by providing rich narratives regarding the timing of trauma-related onset, relationships between trauma and both trauma-related and religious–spiritual content, and trauma and hallucinatory modality. Analysis of participant narratives suggests the central role of localized cultural and sociopolitical influences on onset, phenomenology, and coping and contributes to a growing literature calling for strengths-based, client-driven approaches to working with distressing voices and beliefs that centers the exploration of the personal and social meaning of such experiences including links to life narratives. Findings also underscore the clinical importance of trauma assessment and trauma-informed care.

## Introduction

Traumatic life events (TLEs) have been associated with multiple psychiatric diagnoses, including anxiety disorders, major depression, PTSD, and psychosis (1–3). In a recent epidemiological study, the World Mental Health Survey Consortium found that over 70% of respondents in the general population endorsed exposure to at least one TLE, while 30.5% endorsed four or more lifetime exposures to TLEs (4). Recent meta-analyses have found that persons with TLE exposure are three times more likely to experience psychosis than persons with no past trauma (2) and that TLEs significantly increase the risk for subclinical psychosis (5).

In addition to research linking trauma to psychosis, multiple studies have examined the associations between traumatic experiences and specific symptom domains, including auditory hallucinations (voices), paranoia and delusions (6, 7), as well as non-auditory hallucinations (8, 9). Links between trauma and auditory hallucinations (and to a lesser extent other psychotic symptoms) have also been investigated in combat and non-combat-related PTSD (10–12), borderline personality disorder (13), and dissociative identity disorder (14). Several studies have also sought to unpack the relationship between past trauma exposure and the content of delusions and hallucinations, consistently finding strong direct and/or indirect links between dominant content, themes, and past experiences of adversity (15–18). Structural and community adversity, including migration and ethnic density, are also significant risk factors for psychosis (19, 20).

In spite of a large body of research linking TLEs to psychosis and specific symptom domains, mechanisms of action and the interrelationships between multiple social and environmental risk factors are still not well understood (17, 21–23). Childhood adversity, as well as broader lifetime trauma, tends to be correlated with additional environmental factors, including prenatal insults [such as alcohol exposure during gestation (24), poverty/structural adversity^1^, and personal substance use (25)]. Muenzenmaier et al. (26), have described “complex trauma reactions” triggered by cumulative social adversities and TLEs leading to a broad range of presenting psychotic or psychotic-like symptoms, including dissociation, flashbacks, hallucinations, and paranoid ideation. Recent debates have centered on the differences and overlap between dissociative phenomena, including hallucinations, and psychotic symptoms as they manifest across traditional diagnostic boundaries [e.g., Ref. (15, 27, 28)].

While a number of studies have investigated the role of migration, far fewer have addressed the relationship between race, non-migration-related racism, trauma, and psychosis (29) and found that dissociation only fully mediated the relationship between trauma and psychotic experiences for African-American (not Hispanic or Asian) young adults; while another investigation found that rates of adversity were substantially higher among ethnic minority participants with psychosis and that adversity partially mediated the relationship between ethnicity and psychosis (30). A recent set of analyses utilizing the National Survey of American Life found that multiple types of adversity increased psychosis risk in African-Americans, including neighborhood difficulties and lack of quality educational options (31). To our knowledge, no previous qualitative studies have explored the intersections between trauma, psychosis, and symptom content among African-Americans in the US, although historical and ethnographic work has drawn attention to significant disparities in diagnosis, treatment, and social responses [e.g., Ref. (32, 33)].

To advance our understanding of the complex interactions between forms of adversity as they manifest across the lifespan, psychosis, and symptom content, we undertook a mixed-methods investigation of TLEs and psychosis. Coding and analysis of a separate qualitative sample followed initial analyses of a quantitative sample. A majority of participants were African-Americans, and the qualitative analyses explicitly focused on the experiences of African-Americans participants. Our research questions were as follows:

(1) Is there an association between cumulative exposure, type of TLE, proximity to the traumatic event, and psychosis?
(2) Is there an association between TLEs and clinical symptomology including specific types of delusions and/or hallucinations and in what ways are trauma and past adversity reflected in the form and content of participant’s symptoms?
(3) How does qualitative data further inform our understanding of the complex relationships and patterns of past trauma and adversity and symptoms as they unfold over time?

## Materials and Methods

This study reports the analyses from a novel mixed-methods investigation into the intercept of TLEs and psychosis. The first set of analyses (*n* = 97) focuses on quantitative data from a sample of individuals with and without psychotic disorders recruited from a large urban university medical center, private referrals, and community treatment facilities from January 2013 through January 2015 in Chicago, IL, USA. Participants were recruited using flyers and direct communication with clinical staff regarding study information from a convenience sample. Participants in the quantitative arm of the study were administered standardized measures that are described in detail in Section “Measures Used to Assess Psychosis and TLEs in the Quantitative Sample.”

The second set of analyses (*n* = 34) focuses on a set of individual interviews (*n* = 10) and group interviews (*n* = 24; two focus groups) conducted with individuals reporting experiences of psychosis. The majority of these participants were recruited from a public mental health agency, which serves individuals with serious mental illness and significant, established disability also located in Chicago, Illinois during the same time period. Researchers queried participants about the circumstances surrounding the onset of psychosis; their understanding of the causes and origins of their experiences; and the content, development, and phenomenology of positive symptoms, including the characterological qualities of any voices.

Qualitative interviews and focus groups employed broad, open-ended questions regarding the onset of psychosis or voices; psychosocial context preceding onset; and current symptoms, symptom content, and treatment experiences. Formal diagnostic instruments were intentionally avoided to avoid “clinicalizing” the interviews, and so diagnosis was only recorded through self-report. Background information on past trauma was collected with the demographics. Qualitative data were analyzed using a grounded theory approach (34), which involves focused interview sampling, transcription and summary, coding of data, development of conceptual categories, analytic memoing, and summary of emerging constructs.

### Participants

There were a total of 97 participants in the quantitative study sample, of whom 51 reported present state psychosis consisting of 35 (36%) persons diagnosed with schizophrenia and 16 (17%) diagnosed with bipolar disorder with psychotic features, per consensus diagnosis. Consensus diagnosis was determined by reviewing all research data and collateral information by the study personnel that included an Attending Physician, Psychologists, and a Mental Health Nurse Practitioner. Inclusion criteria for the study included participants between the ages of 21 and 60. The clinical sample must have met criteria for schizophrenia or bipolar disorder/psychosis. A group of 46 (47%) non-clinical controls, with no history of DSM-IV-TR Axis 1 diagnosis per the SCID, were also recruited. Exclusion criteria for both groups included current substance dependence, seizure disorders, and neurological conditions. All participants, clinical and non-clinical, were reimbursed equally for their time and transportation. Demographic characteristics for the sample (Table 1) and clinical metrics were obtained at the study evaluation.

**Table 1:** **Demographic characteristics of quantitative sample**.

Demographic measure	Schizophrenia (*n* = 35)		Bipolar disorder with psychosis (*n* = 16)		Non-clinical control (*n* = 46)		*p* Value
**Sex (male/female)**	19/16		7/9		16/30		n.s.
**Race**							0.02*
African-American	26		13		20		
Caucasian	3		3		11		
Hispanic	4		0		5		
Other	2		0		10		

	**Mean**	**SD**	**Mean**	**SD**	**Mean**	**SD**	***p* Value**

**Current age**	42	12.31	47	12.19	38	12.27	0.04*

*n.s., not significant*.

*p < 0.05.

There were a total of 34 qualitative study participants, all of whom reported either schizophrenia spectrum diagnosis or bipolar disorder with psychotic features. The majority also reported past or comorbid diagnoses of PTSD, dissociative identity disorder, anxiety disorders, and/or major depression. All participants described current hallucinations and/or delusions during interviews and groups. Demographics are listed in Table 2. The majority of participants were African-Americans (23/34; 68%). All participants signed consent forms and agreed to be audiotaped for research purposes. Interviews and focus groups lasted from 1.5 to 3 h and were led by an interviewer–facilitator with personal experience of psychosis, which was intentionally disclosed as a part of the interview process.

**Table 2:** **Demographic characteristics of qualitative sample**.

Demographic measure	Focus groups percent (*n*)	Individual interviews percent (*n*)
**Sex (male/female)**	16/8	4/6
**Race**		
African-American	18	5
Caucasian	2	3
Hispanic	2	0
Other/mixed	2	2

	**Mean**	**Mean**

**Current age**	41.4	49.1

### Measures Used to Assess Psychosis and TLEs in the Quantitative Sample

Multiple clinical measures were employed to examine the relationship between TLEs and psychosis. The evaluation of forms of delusions and hallucination was based on the SCID and scored for lifetime exposure as absent (score of “1”), subthreshold (“2”), and threshold or present (“3”). The SCID includes an assessment of forms of delusions (referential, persecutory, grandiose, somatic, religious, guilt, jealous, erotomanic, control, thought insertion, thought withdrawal, and thought broadcasting) and hallucinations (auditory, visual, tactile, gustatory, and olfactory) (35).

The Life Events Checklist consists of 17 items that measure exposure to various TLEs (36). The items consisted of exposure to (1) natural disaster, (2) fire or explosion, (3) transportation accident, (4) serious physical accident, (5) exposure to toxic substance, (6) physical assault, (7) assault with a weapon, (8) sexual assault, (9) other unwanted or uncomfortable sexual experiences, (10) combat or exposure to a war zone, (11) captivity, (12) life-threatening illness or injury, (13) severe human suffering, (14) sudden violent death, (15) sudden unexpected death of someone close, (16) serious injury, and (17) harm or death you caused to someone else or any other very stressful event. For each event, participants were asked to select one or more event by checking all that applied along the following continuum: (a) happened to me, (b) witnessed it, (c) learned about it, (d) not sure, and (e) doesn’t apply.

The PANSS was scored along a continuum of severity between one (asymptomatic) to seven (extreme symptom severity). Analysis was conducted *via* data reduction strategies guided by prior empirical studies of symptom domains assessed by the PANSS (37). Scores were calculated for five factors: positive symptoms (delusions, grandiosity, suspiciousness/persecution, and unusual thought content), negative symptoms (blunted affect, emotional withdrawal, poor rapport, passive/apathetic social withdrawal, lack of spontaneity and flow of conversation, and active social avoidance), cognitive disorganization (conceptual disorganization, difficulty in abstract thinking, mannerisms and posturing, disorientation, and poor attention), excitement (excitement, hostility, tension, and poor impulse control), and depression (somatic concern, anxiety, guilt feelings, depression, and preoccupation). Second, PANSS items that have been shown to identify related symptom domains in cluster analyses that assess anergia (blunted affect, emotional withdrawal, motor retardation, and disorientation), thought disturbance (conceptual disorganization, hallucinatory behavior, grandiosity, and unusual thought content), activation (excitement, hostility, tension, poor impulse control), paranoia (suspiciousness/persecution, hostility, and uncooperativeness), and prosocial (active social avoidance, passive social withdrawal, emotional withdrawal, suspiciousness\persecution, stereotyped thinking, and hallucinations) were also obtained. Items were pooled in this way based on previous factor analytic findings (38, 39). Coefficient alpha, for interrater reliability, was between 0.83 and 0.87.

### Data Analyses

Demographic data were analyzed using chi-square tests and analyses of variance (ANOVA). For ANOVAs that yielded significant results (alpha level <0.05), Newman–Keuls *post hoc* tests were used to identify significant pairwise group differences. We conducted a binary logistic regression to examine the probability that cumulative TLEs increased the likelihood of psychosis. An independent sample *t*-test was conducted to compare the association of TLEs and aspects of psychosis. Bivariate spearman correlations were conducted to determine separate associations between TLEs and clinical symptomology with particular attention to forms of delusions and hallucinations between persons with psychosis and the non-clinical group. All quantitative data were analyzed using SPSS software version 24.

For the qualitative component of the project, *a priori* codes were generated following the associations identified as significant and/or unexplained in the quantitative analyses. For example, we explicitly sought to identify and unpack cumulative associations between diverse traumatic events and broader adversity, a topic that the trauma measure utilized in the quantitative study could not address. We also coded the content of participant’s symptoms, looking for associations between past adversity, trauma, and the themes of voices and/or unusual beliefs. Transcripts were independently coded at different time points to establish reliability.

## Results

### Quantitative Analyses

Demographic characteristics of the quantitative sample are presented in Table 1. Group comparisons revealed a significant difference in race between diagnostic groups, with a larger proportion of African-American participants in the schizophrenia group relative to both the bipolar/psychosis and non-clinical groups (χ^2^ = 15.38, df = 6, *p* < 0.02). In total, just over three quarters (77%) of clinical participants were African-American. We also found a significant difference in age across diagnostic groups, showing that participants with bipolar/psychosis were older than participants with schizophrenia and non-clinical controls (*F*_2,94_ = 3.282, *p* < 0.04). There was no significant sex difference between diagnostic groups or the non-clinical group.

When comparing persons with psychosis (*n* = 51) to the non-clinical group (*n* = 46), there were no group differences in the overall total score of TLEs exposure (*t*_95_ = 0.43, *p* = 0.67). However, we did find a significant group difference in the exposure of TLEs that “Happened” to the individual (as opposed to those who “Witnessed” or “Learned” about the event), showing that as lifetime cumulative TLEs exposures that “Happened” to the individual increased, the more likely the individual was to exhibit symptoms of psychosis (*t*_95_ = 2.42, *p* = 0.02). We also examined differences in exposure of TLEs that “Happened” and race and found a significant increase in TLEs that “Happened” in the African-Americans sample (*F*_3,93_ = 3.03, *p* = 0.03) (M = 4.27, SD = 3.19). In addition, we found a significant effect of cumulative TLEs on psychosis (OR = 1.174; *p* < 0.02). Thus, as the number of TLEs increased by one unit, the likelihood of the presence of psychosis also increased.

Spearman’s correlations between the number of exposures of TLEs that “Happened” to the individual and clinical symptomology as measured by the PANSS are presented in Table 3. We found a positive correlation between lifetime cumulative TLEs that “Happened” to the individual and several, but not all, PANSS symptom domains, showing that as the number of TLE exposures that “Happened” increased, the severity of clinical symptomology increased. There was a positive correlation between the number of TLEs and the following PANSS factor and composite scores: positive (*r* = 0.241, *n* = 97, *p* = 0.02), excitement (*r* = 0.302, *n* = 97, *p* = 0.003), depression (*r* = 0.310, *n* = 97, *p* = 0.002), thought disorder (*r* = 0.216, *n* = 97, *p* = 0.03), activation (*r* = 0.266, *n* = 97, *p* = 0.008), and paranoia (*r* = 0.224, *n* = 97, *p* = 0.03). However, there was no correlation between the number of TLEs and negative, cognitive, anergia, or prosocial symptom domains.

**Table 3:** **Correlations between “Happened” traumatic life events (TLEs) and clinical symptomology**.

	Variables	Life Events Checklist (LEC)
1.	LEC	~
2.	PANSS positive	0.241*
3.	PANSS negative	0.113
4.	PANSS cognitive	0.180
5.	PANSS excitement	0.302**
6.	PANSS depression	0.310**
7.	PANSS anergia	0.131
8.	PANSS thought disorder	0.216**
9.	PANSS activation	0.266**
10.	PANSS paranoia	0.224*
11.	PANSS prosocial	0.139

*Spearman’s correlations between “Happened” TLE and forms of delusions*.

*p < 0.05.

**p < 0.01.

We also examined the correlations between the number of exposures of TLEs that “Happened” to the individual and hallucinations (Table 4). With the exception of gustatory hallucination, significant positive correlations were found between TLCs exposure that “Happened” to the individual and all hallucination modalities: auditory hallucinations (*r* = 0.196, *n* = 97, *p* = 0.05), commenting hallucinations (*r* = 0.254, *n* = 97, *p* = 0.01), conversing hallucinations (*r* = 0.255, *n* = 97, *p* = 0.012), visual hallucinations (*r* = 0.257, *n* = 97, *p* = 0.011),; tactile hallucinations (*r* = 0.420, *n* = 97, *p* = 0.000), and olfactory hallucinations (*r* = 0.296, *n* = 97, *p* = 0.003).

**Table 4:** **Correlations between “Happened” TLEs and hallucinations**.

	Variables	Life Events Checklist (LEC)
1.	LEC	~
2.	Auditory hallucinations	0.196*
3.	Voices commenting	0.254**
4.	Voices conversing	0.255**
5.	Visual hallucinations	0.257**
6.	Tactile hallucinations	0.420***
7.	Gustatory hallucinations	0.125
8.	Olfactory hallucinations	0.296**

*Spearman’s correlations between “Happened” TLE and hallucinations*.

*p < 0.05.

**p < 0.01.

***p < 0.001.

Correlations between the number of TLEs that “Happened” to the individual and forms of delusions are reported in Table 5. We found positive associations between the number of TLE exposures that “Happened” to the individual and 12 specific forms of delusions, indicating that as the number of TLE exposures that “Happened” increased, the more likely they were to experience specific forms of delusions. However, only associations between the number of TLEs and religious delusions (*r* = 0.196, *n* = 97, *p* = 0.05) and delusions of control (*r* = 0.196, *n* = 97, *p* = 0.05) were statistically significant, with an additional trend in persecutory delusions (*r* = 0.191, *n* = 97, *p* = 0.06). There was no correlation between the number of TLEs that “Happened” and delusions of reference, grandiosity, somatic, guilt, jealous, erotomanic, thought insertion, thought withdrawal, and thought broadcasting.

**Table 5:** **Correlations between “Happened” TLEs and delusions**.

	Variables	Life Events Checklist (LEC)
1.	LEC	~
2.	Delusions of reference	0.145
3.	Persecutory delusions	0.191
4.	Grandiose delusions	0.156
5.	Somatic delusions	0.109
6.	Religious delusions	0.196*
7.	Delusions of guilt	−0.023
8.	Jealous delusions	−0.025
9.	Erotomanic delusions	−0.013
10.	Delusions of control	0.196*
11.	Delusions of thought insertion	0.147
12.	Delusions of thought withdrawal	0.092
13.	Delusions of thought broadcasting	0.164

*Spearman’s correlations between “Happened” TLE and delusions*.

*p < 0.05.

Given the highly correlated findings between TLEs in both religious delusions and tactile hallucinations, we conducted an independent sample *t*-test to explore associations between specific TLEs involving physical and/or sexual assault, unwanted or uncomfortable sexual experiences, and religious delusions and tactile hallucination. Interestingly, we found a positive relationship between tactile hallucinations and physical assault (*t*_95_ = 3.95, *p* = 0.000), sexual assault (*t*_95_ = 4.65, *p* = 0.000), and unwanted or uncomfortable sexual experiences (*t*_95_ = 3.18, *p* = 0.003). We also found a positive association between religious delusions and unwanted or uncomfortable sexual experiences (*t*_95_ = 1.98, *p* = 0.05).

### Qualitative Analyses

Interviews and focus groups were transcribed verbatim, and a modified grounded theory approach was used to identify themes related to the intersections of race, trauma, and psychosis. Transcripts were comprehensively coded and recoded after a 3-month interval to establish reliability (kappa > 0.90). Findings revolve around four major thematic umbrellas: (1) developmental relationships between multiple, intersecting adverse experiences; (2) variations in the timing of onset related to trauma; (3) trauma, spirituality, and religious symptom content; and (4) trauma and hallucinatory modalities.

#### Developmental Relationships between Multiple Adversities

Virtually all participants, and all African-American participants, described some form of trauma and, in the majority of cases, multiple forms of adversity, including structural discrimination and racism that interacted synergistically over the course of childhood, adolescence, and early adulthood. Many participants also reported family members with serious mental illness, poverty, unstable home environments, neglect, verbal and/or physical abuse, and disruption of attachment relationships.

- “The voices that I heard was bad ones for the most part. I came from an abusive background, meaning that my family was abusive physically, emotionally and sexually. I was raped and I just heard all these voices. That’s why the doctors thought initially I had schizophrenia.”
- “I would get very mad at my mother for being gone. For having passed away. My mother put me in the hospital kind of like my dad. He was in the same hospital, too. He had a mental illness. My father was also in jail. He was put into a hospital in the past too. He was a patient there. He had voices. He often talked to himself and I would wonder why is he talking to himself? Who is he talking to? My dad heard voices. I caught him in that, too, many times talking to himself. Sometimes I would go to church and people were talking like they heard voices. Sometimes I would see my father talking about [voices] and ready to beat me up and he got very angry. He would say, ‘Just get out of here. Go on back home.’ He would just get really agitated….”
- “Since I was like 12, 13 years old, I used to talk to myself, caught my voices. I didn’t know what it was then. I assumed it was me, cuz I heard it very early. I came introverted somewhat cuz there’s a lotta violence in the streets. I was more of a homebody. I think, for me being a homebody, that’s when the voices got brought up. I just had to entertain myself with the voices and talking.”

Narratives of gangs, substance use, and drug trafficking were often woven throughout. One participant described learning (as an older teen) that his mother had been drinking heavily while pregnant with him, while others described parents with severe addictions throughout their childhoods, as well as parents incarcerated for drug-related offenses. Still others indicated pressure from siblings or peers to start using drugs, as well as more complicated entanglements of money, gangs, and drug use.

- “The kids I hanging with. They got me on drugs. I think it was a negative factor, negative influence on my life, the kids I was with. Other than that, I probably wouldn’t be doing drugs. Yeah, definitely drugs is a factor in [my diagnosis].”
- “I was just getting angry, depressed, sad. My parents, I heard my mother say—she was drinking. She was drinking when she […] was pregnant with me. I heard my father was on my marijuana. Other than them, my mental illness is from both sides of my family, my father and mother’s side. They both have mental illness. I’m just sure that I get—I see it in my auntie, too, on my mother’s side. I see some mental illness with her and everything. It runs in the family.”
- “My mother, she was a heroin addict. I had a problem with her ‘cause people wanna take advantage of her. That’s where most my problems start. They pissed me off ‘bout my mother. I started stickin’ up, stealin’ to start supplyin’ my mother’s heroin so she wouldn’t have to go out there in the streets. That caused conflict between me and my brother. Me and him got in a fight one time. The second time I shot him in the foot. All that’s to say is that I never had no chance at life—childhood or life.”

Among African-American men, a strong subtheme was bullying and/or verbal abuse (often from male members of the household or male peers) related to perceptions of effeminate behavior. For example, several participants reported having been bullied due to perceived homosexuality. Several participants reported responding to such accusations by engaging in stereotypically masculine behaviors such as joining a gang or drinking, while others, publicly or in private, embraced gender minority identities, with one engaging in same-sex sex work as a teen.

- “I was bullied, too, in high school and grammar school. I was bullied. People messing with me and everything, beating me up. I got jumped on by some gang bangers. Used to pick on me a lot like I don’t know how to defend myself or something. Pick on me and everything. I know how to protect myself, though. I ain’t gonna let too many people try to mess me up. My father said that I was gay. He said, ‘People think you gonna be gay cuz you don’t have many women around. [But] I wasn’t gay…. I’m not gay.’”

The above experiences virtually all unfolded in racially segregated neighborhoods and housing projects with high rates of poverty, gang violence, and limited access to quality job and/or secondary education. About half the participants remained in such neighborhoods and explicitly foregrounded the risks of violence in their communities as a major factor in their experiences of ongoing treatment and recovery. Just 2 weeks before, a focus group held at a Chicago South Side drop-in center, for example, participants noted that, only a few blocks away at a public park, over a dozen community members (including children) had been killed or seriously injured in a day-time drive-by shooting.

#### Timing of Trauma-Related Onset

In discussing the relationship between traumatic events and the onset of psychosis, most participant accounts fell into one of two categories: childhood onset of voices in the midst of acute traumatic experiences (e.g., sexual abuse) and older adolescent or adult onset following a series of adversities (as well as drug or alcohol use) but that was nevertheless temporally disconnected from any *single* traumatic event.

The content and characteristics of voices arising during acute traumatic events in childhood were generally much more likely to mirror real-life abusive figure(s). For instance, voices that began during episodes of severe sexual or physical abuse were typically verbally abusive, telling participants that they “deserved what [they] got,” were “whores” or “sluts,” would never amount to anything and/or never succeed.

- “There’s a lot of voices that I hear. All grown men saying the same thing over and over. I’m no good, I’m worthless. Kill yourself. Just repeatedly over and over and over and over by men.”
- “…they keep telling me to kill myself. I’m no good. I’m worthless. To kill myself repeatedly.”
- “A lot of negative remarks, they ain’t gonna amount to being nothing, they gonna grow up to be nothing, you’re a failure, and all that stuff.”

One participant with trauma-onset voices described her voices as “glued to your experience through childhood experience.” In spite of hearing voices from an early age—typically beginning in early to mid-childhood within this subgroup—most participants reported not receiving a diagnosis of (or treatment for) a psychotic disorder until a much later time point. It was generally ambiguous whether these later diagnoses were exclusively tied to voices, or in fact stemmed from the addition of later onset symptoms and/or functional disability.

For those with later onset and multiple forms of adversity and associated risk taking (substance use, drug trafficking, and participation in street gangs, as described above), connections between psychotic symptoms and particular experiences were less clear—e.g., it was far less common for participants to report voice or symptom contact that amplified or re-played a particular event and associated interpersonal exchanges (such as the messages of an abusive figure). Themes consistent with participants’ lives and broad experiences of individual and community adversity and discrimination were nevertheless common, for example, delusions involving gangs or pimps or demonic voices tempting participants to use drugs or engage in illegal activities.

#### Trauma and Religious–Spiritual Content

The majority of participants described multiple entanglements between religious faith, spiritual beliefs, and their experiences of psychosis and adversity. Examples ranged from distinct voices that reflected demonic forces, or a G-d or Jesus-like messianic figure, ostensible delusions of reference involving messages from G-d or the devil, automatic thoughts related to participants’ addictions, and perceived temptations as attributable to the devil while automatic protective thoughts were attributed to G-d; and the involvement of pastors or other religious figures who helped participants make sense of their experiences and distinguish between those that were divine versus demonic in origin.

- “Well, there are voices from above. I label it as G-d. It’s a noise, but it’s a feeling of ‘I must something.’ It is like a voice of something talking. Sometimes it’s sort of like an amen kind of a noise. Like a church music kind of noise. That kind of noise. There are voices within that.”
- “I think they’re demons. I think that G-d talks to you and I think that demons talk to you. When I was drinking and using drugs, they was always telling me to kill myself. That I wasn’t worthy, that I didn’t amount to nothing. When I gave my life to G-d and turned my life over, the devil still tried to come at me. I would hear his voice, but I also read the bible and I believe in the word of G-d. I had to choose G-d’s word over what the devil was tellin’ me. The bible tells me that the devil is a liar. Whatever he’s tellin’ me that’s negative, he’s tellin’ me to kill myself, that’s not something that G-d would want me to do.”
- “When I go to church and all, they teaches me to tell me why the voices are talking like that. [*Interviewer*: Your pastor at church does?] Yeah, tells me not to listen to the voices telling me to hurt yourself.”
- “I said that I’ll be listening to the preacher preaching, and he’s telling me which one is—why you shouldn’t listen to the bad side. The bad side try to get you to hurt yourself. He be talking about teaching ’em [voices] how to do good things, too, but he be telling why the bad things be happening, like getting jumped on or getting beat up a lot. They tell you what spirit that is telling you to hurt yourself.”

Trauma was often tied to these experiences in both direct and indirect ways. For example, demonic “temptations” (whether communicated through voices or non-auditory messages) often involved risk behaviors deeply interwoven with neighborhood poverty and a lack of supports and access to child and family services such as drug use, drug trafficking, gang involvement, and survival sex. Positive voices, even when not explicitly religious or spiritual in nature, were not infrequently described as forces or entities, which would protect the participant against adversity or violence or to reassure them of their core morality or humanity in spite of “immoral” activities and events they had been exposed to (or previously participated in).

- “One time I go outside at 2:00 a.m. Somethin’ tells me that somethin’ ain’t right. Like you talkin’ to me like it’s a warning sign. Like you talkin’ to me, that’s the way—that’s how I hear it. Like you talkin’ to me. I changed my mind. I’m not gonna catch the bus. I turned around and went upstairs. Where I live there’s a park across the street. One of the bus stops is right there. About 2-min long up the steps I heard some shootin’. As I look out the window, I saw a body layin’ down and a couple guys runnin’. The very same bus stop I was gonna catch the bus at. That’s why I say it’s a good voice and a bad voice. The voice that I heard, I feel it was comin’ from G-d. If it was an evil voice, I feel that I wouldn’t got that notice.”

Several participants whose narratives involved accusations and/or internalized concerns about sinful behavior and/or stigmatized gender or sexual identit(ies) described various uses of religious faith or spiritual practice to address their concerns or reassure themselves that they were (in fact) good, ethical people.

- “Jesus [described as a voice earlier in the interview] reassures me…that He loves everyone. Regardless of sexual orientation.”
- [Reporting what her voices say]: “I get, ‘That’s not the right thing to do, Carol [pseudonym].’” *[Pause]* “You can get through this day without thinking about going back and using drugs.”

#### Trauma and Hallucinatory Modality

While participants most frequently described, and spent the most time discussing, ostensible delusions (particularly paranoia) and auditory hallucinations (“voices”), altered perceptual or somatic experiences in other modalities were common. Participants’ descriptions generally revolved less around literal “hallucinatory” experiences than they did around more existential alterations of experience and/or “felt presences,” which tended to be subtler and harder to describe. In many cases, participants reported that such presences represented ghosts or spirits, generally of deceased loved ones.

- “A smell like, let’s see—maybe it tastes like you wanna drink some water cuz I’m dizzy. Almost like a shocking feeling, but the smell it’s like maybe more like a taste. Maybe it smells like air, like air.” [*Q*: In some way that’s different from the air that’s already there?] “Right, it’s not the same. It’s different.”
- “I can see my [deceased] aunt and my uncle and you see, I see them. Yeah, I see a lot of people up there when I look up [toward the voices].”
- “I feel like, like it’s a lifting, sort of like a lifting feeling, like of something being lifted.”
- “For me it’s more like a feeling, too. Like something that makes me turn my head. Like something that’s there that I—almost like a buzz or a tone or something.”

A number of participants connected non-auditory–verbal experiences directly to G-d or spirituality. For example:

- “It’s bothersome and there’s noise, but then it’s like this singing like this noise, and I feel like G-d, you know, G-d makes these noises [‘voices’].”

In contrast to the previous themes, direct links between these non-verbal experiences and trauma were unclear, and no participant in this sample explicitly described non-auditory experiences as sexual or violent in nature.

## Discussion

The urban experience for African-Americans living in segregated neighborhoods and/or housing projects often involves exposure to high rates of poverty, gang violence, and limited access to quality secondary education and job opportunities (40, 41). Increased exposure to TLEs tends to be more frequent in low SES and racial minorities groups (42, 43). In comparison to Caucasians, African-Americans have reported increased exposure to violent assaults (44, 45) including gun-related violence (46, 47). Although racial disparities in access and outcomes are well documented [e.g., Ref. (48, 49)], relatively little empirical research has focused on the complex relationships between trauma and the subjective experience and phenomenology of psychosis, including symptom content, within specific ethnic/racial/cultural minority communities [for exceptions, see Ref. (33, 50)].

The mixed-methods data reported here replicate existing findings in identifying strong and significant links between cumulative trauma exposure and psychosis that cut across traditional diagnostic boundaries (2, 3, 51, 52). We extend this literature by demonstrating that these findings hold in our predominantly African-American US sample, where the relationship between TLE and psychosis may be particularly relevant due to higher likelihood of TLEs among ethnic minorities. Increased and more nuanced understanding of the role that such environmental factors play in the development of psychotic disorders helps parse the heterogeneous etiology of these illnesses and possibly points toward more personalized treatment conceptualizations.

Beyond demonstrating a general link of greater TLEs and psychosis, we found specific types of psychosis symptoms associated with TLEs, including multiple types of hallucinations, religious delusions, and delusions of control. We further found that tactile hallucinations and religious delusions were significantly correlated with history of unwanted sexual experiences and, for tactile hallucinations, with past physical and sexual assault. We discuss these findings and their implications in the sections that follow.

Our qualitative analyses were designed to further unpack, potentially confirm, and deepen the findings from the quantitative study. The qualitative findings underscore the complex and synergistic relationships between multiple forms of individual trauma (including bullying, harassment, and abuse) as they unfold against a backdrop of racial segregation, poverty, drug trafficking, gang violence, and neighborhood disadvantage. In addition, we report multiple ways in which both individual-level and neighborhood-level themes and dynamics are reflected in the form and content of both voices and unusual beliefs. For example, we found that an array of themes related to participants’ childhood experiences and associated attempts to cope with these experiences were reflected in both the content of voices and subject’s interpretations of their meaning and significance.

### Cumulative Exposure, Type, and Proximity to the Traumatic Event and Psychosis

National comorbidity studies and meta-analysis have reported a potentially causal relationship between cumulative TLEs exposure and psychosis (26, 53, 54). Our data are consistent with other studies that show that the overall lifetime TLE exposure is significantly higher in the African-American population (47, 55, 56). In turn, our finding that cumulative TLE exposures that “Happened” to the individual increased relative to reporting psychotic symptoms is consistent with existing research, highlighting the likelihood of multiple exposures amplifying the risk of psychosis beyond individual stressors alone (5, 25, 57). For example, analysis of the United States National Comorbidity Survey (NCS; *n* = 5,782) and the British Psychiatric Morbidity Survey (BPMS; *n* = 8,580) found that after adjusting for demographic confounds, substance use, and depression, experiencing two TLEs increased psychosis risk by 3.37 times (NCS) and 4.31 times (BPMS), respectively, compared to 30.16 times (NCS) and 192.97 times (BPMS) for individuals reporting five TLEs (58).

The nature of the relationship between collective trauma and/or socioenvironmental adversity and psychosis indicates that the urban environment may increase the likelihood of exposure to TLE in persons who later develop psychosis (59). Our qualitative findings underscore the synergistic effects of both individual- and neighborhood-level adversity, with both psychological and biological components, including exposure to illicit substances (prenatal and during childhood/adolescence) and chronic background stress. We suggest that future research needs to more explicitly model both biological and (ongoing) psychological mechanisms and associated (adaptive or maladaptive) coping into early adulthood. Participant narratives also suggest that neighborhood adversity, particularly threat of gang-related violence, may be an important maintaining factor for both paranoid beliefs and voices.

### TLEs and Participant’s Symptoms in Form and Content

Associations between trauma and delusions have been reported in multiple studies (23, 57, 60). A study conducted by Scott et al. (12), examining the association between trauma and delusions found that persons who endorsed any type of delusion were significantly more likely to have been exposed to a traumatic event and that as the exposure to trauma increased, the relative risk of experiencing delusions increased significantly. Exposure to an urban environment has been shown to increase anxiety, negative belief about others, and jumping to conclusions in persons with persecutory delusions when compared to a non-clinical group (61). Interactions amongst discrimination, deprivation, stress, mistrust, social inequality, and lack of social support were proposed as predictors of both affective and non-affective psychosis (62). Likewise, auditory hallucinations across diagnoses have been strongly linked to childhood adversity, particularly sexual abuse (63, 64).

In line with previous research, we also found multiple direct and/or indirect emotional and thematic links between adversity exposure and the content of voices and delusions, including voices that mimicked the sentiments of abusive figures, and paranoia that reflected community contexts characterized by poverty, gang, police activity, and near constant background threat of violence (15, 16, 18). Rather than finding that voices and delusional beliefs predominantly reflected negative trauma-linked content, many of our participants reported positive and/or protective content, including voices perceived to originate from G-d, Jesus, or protective spirits, findings which are consistent with Jones et al. (65). Our finding that several participants reported voices as a source of comfort and support also highlights the need for sensitivity amongst healthcare workers in not treating hallucinations as unilaterally negative experiences, which need to be eradicated.

### TLEs, Psychosis, and Spiritual Experiences

In the existing literature, religious beliefs have been reported as a source of strength, comfort, and encouragement in managing psychiatric difficulties related to traumatic events (66, 67). Religion or spirituality may also provide a framework to understand or bring meaning to the individual who experienced a TLE (68, 69). Religious practices such as prayer or meditation, worship, and participation in religious services can engender hope and increased social support among individuals with serious mental illness (70–72). While not universal, religious/spiritual explanations of psychotic experiences, religious themes, and/or content (such as hearing the voices of G-d), and faith-based coping and healing practices (including explicit discussions regarding the navigation of voices with pastors or preachers) were common. For many participants, positive religious or spiritual beliefs, including those entangled directly in their voices and psychotic experiences, were described as offering advice, guidance, moral reassurance, and/or fortification against temptations or demonic intrusions. For at least some clients, these temptations or intrusions (for example, commands such as “take drugs,” “shoot him,” “have sex,” or delusions involving similar themes) were directly associated with past adversity.

### Relationship and Patterns of Past Trauma and Symptoms over Time

The causative evidence of the association between trauma and psychosis is the strongest for the manifestation of hallucinations (73). Experiencing trauma has been shown to increase the likelihood of verbal hallucination fivefold (74). The phenomenological associations between trauma and hallucinations have shown that hallucinations with content related to trauma are not only found in psychosis they may actually shape the themes of the hallucinatory experience (16, 73, 75). In a recent systematic literature review of studies investigating voices, the association between trauma and voices has been explored in multiple realms including phenomenology, causal link, neurobiological hypotheses, and treatment interventions (76). Much of the research emphasis has focused on the associations between trauma and verbal hallucinations with much less emphasis between trauma and other types of hallucinations, including tactile or olfactory (77). Our research examined the association of trauma and all forms of hallucinations. Interestingly, the strongest association was found between TLE’s that “Happened” and tactile hallucinations, although there was a positive correlation between TLE and most types of hallucinations. Our qualitative data further link the association between a specific traumatic event such as sexual assault and the onset of psychosis and draws attention to a potentially traumatogenic subgroup of patients whose voices began in the midst of acute trauma in childhood but were later diagnosed with a psychotic disorder, and a subgroup who, in spite of significant trauma, did not develop psychosis until early adulthood. This finding further supports the clinical need of evaluating current and past trauma throughout the lifespan as symptoms associated with a TLE may occur during or in close time proximity the event or may not manifest until much later in life (78).

There are limitations of this study in that both quantitative and qualitative datasets reported here are cross-sectional and can only establish correlations and perceived causal connections rather than empirical causality. In addition, our sample was predominantly African-Americans living in a particular, notoriously segregated urban environment; our qualitative analyses are meant to deepen our understanding of the interplay of trauma, psychosis, and spirituality within a particular group and associated sociogeographic context, not to generalize. We also had no mechanism for verifying TLE, although it should be noted that retrospective accounts of adversity amongst psychosis populations have consistently been shown to be reliable and valid and are more likely to underreport than overreport TLEs (79–81).

Finally, our findings echo the extant literature in foregrounding the importance of childhood adversity, neighborhood characteristics, and cumulative adversity with response to both the epidemiology of psychosis and the process of recovery and healing. Experiences described by qualitative participants were far from unilaterally negative, and participants consistently linked the content of symptoms to an array of life events and challenges. Taken together, these findings foreground the importance of trauma assessment and conversations aimed at understanding the role that traumatic experiences have played in clients’ lives, in the genesis of their mental health challenges, and in the content of these experiences. From a public health perspective, they add further fuel to calls for both research and preventative interventions aimed at addressing the negative impacts of childhood structural adversity and neighborhood disadvantage.

## Ethics Statement

The quantitative analysis was conducted and approved by University of Illinois at Chicago Institutional Review Board (IRB). The University of Illinois at Chicago Institutional Review Board approved the study, and signed consent was obtained from all participants in accordance with the Declaration of Helsinki before initiation of study procedures. The qualitative analysis by DePaul University’s Institutional Review Board where two of the researchers were employed at the time the study was approved and data were collected.

## Author Contributions

CR, NJ, and RS designed the study. CR and NJ collected the data. CR, NJ, KC, and RS developed the methodology and performed the analysis. CR, NJ, EL, KC, MS, JM, SK, and RS wrote, edited, and approved the final version of the manuscript.

## Conflict of Interest Statement

The authors declare that the research was conducted in the absence of any commercial or financial relationships that could be construed as a potential conflict of interest.

## Footnotes

^1^Jones N, Godzikovskaya J, Zhao Z, Vasquez A, Davidson L. Intersecting disadvantage: unpacking sub-optimal outcomes within early intervention in psychosis services. *Early Interv Psychiatry* (under review).

## References

[1] Kendler KS Karkowski LM Prescott CA. Causal relationship between stressful life events and the onset of major depression. Am J Psychiatry (1999) 156(6):837–41. 10.1176/ajp.156.6.837 10360120

[2] Beards S Gayer-Anderson C Borges S Dewey ME Fisher HL Morgan C. Life events and psychosis: a review and meta-analysis. Schizophr Bull (2013) 39(4):740–7. 10.1093/schbul/sbt065 23671196 PMC3686461

[3] Russo DA Stochl J Painter M Dobler V Jackson E Jones PB Trauma history characteristics associated with mental states at clinical high risk for psychosis. Psychiatry Res (2014) 220(1–2):237–44. 10.1016/j.psychres.2014.08.028 25200190 PMC4218920

[4] Benjet C Bromet E Karam EG Kessler RC McLaughlin KA Ruscio AM The epidemiology of traumatic event exposure worldwide: results from the world mental health survey consortium. Psychol Med (2016) 46(2):327–43. 10.1017/S0033291715001981 26511595 PMC4869975

[5] Varese F Smeets F Drukker M Lieverse R Lataster T Viechtbauer W Childhood trauma increases the risk of psychosis: a meta-analysis of patient-control, prospective- and cross-sectional cohort studies. Schizophr Bull (2012) 38(4):661–71. 10.1093/schbul/sbs050 22461484 PMC3406538

[6] Bentall RP Wickham S Shevlin M Varese F. Do specific early-life adversities lead to specific symptoms of psychosis? A study from the 2007 the adult psychiatric morbidity survey. Schizophr Bull (2012) 38(4):734–40. 10.1093/schbul/sbs049 22496540 PMC3406525

[7] Bentall RP de Sousa P Varese F Wickham S Sitko K Haarmans M From adversity to psychosis: pathways and mechanisms from specific adversities to specific symptoms. Soc Psychiatry Psychiatr Epidemiol (2014) 49(7):1011–22. 10.1007/s00127-014-0914-0 24919446

[8] Longden E Sampson M Read J. Childhood adversity and psychosis: generalised or specific effects? Epidemiol Psychiatr Sci (2016) 25(4):349–59. 10.1017/S204579601500044X 26156083 PMC7137611

[9] Solesvik M Joa I Larsen TK Langeveld J Johannessen JO Bjornestad J Visual hallucinations in first-episode psychosis: association with childhood trauma. PLoS One (2016) 11(5):e0153458. 10.1371/journal.pone.0153458 27144681 PMC4856312

[10] David D Kutcher G Jackson EI Mellman TA Psychotic symptoms in PTSD. New Research Program and Abstracts. American Psychiatric Association Annual Meeting (1997).

[11] Holmes DS Tinn LW The problem of auditory hallucinations in combat PTSD. Traumatology (1995) 1(2). 10.1177/153476569500100201

[12] Scott J Chant D Andrews G Martin G McGrath J. Association between trauma exposure and delusional experiences in a large community-based sample. Br J Psychiatry (2007) 190:339–43. 10.1192/bjp.bp.106.026708 17401041

[13] Kingdon DG Ashcroft K Bhandari B Gleeson S Warikoo N Symons M Schizophrenia and borderline personality disorder: similarities and differences in the experience of auditory hallucinations, paranoia, and childhood trauma. J Nerv Ment Dis (2010) 198(6):399–403. 10.1097/NMD.0b013e3181e08c27 20531117

[14] Dorahy MJ Corry M Shannon M Macsherry A Hamilton G McRobert G Complex PTSD, interpersonal trauma and relational consequences: findings from a treatment-receiving northern Irish sample. J Affect Disord (2009) 112(1–3):71–80. 10.1016/j.jad.2008.04.003 18511130

[15] Corstens D Longden E The origins of voices: links between life history and voice hearing in a survey of 100 cases. Psychosis (2013) 5(3):270–85. 10.1080/17522439.2013.816337

[16] Hardy A Fowler D Freeman D Smith B Steel C Evans J Trauma and hallucinatory experience in psychosis. J Nerv Ment Dis (2005) 193(8):501–7. 10.1097/01.nmd.0000172480.56308.21 16082293

[17] Raune D Bebbington P Dunn G Kuipers E. Event attributes and the content of psychotic experiences in first-episode psychosis. Psychol Med (2006) 36(2):221–30. 10.1017/S003329170500615X 16336724

[18] Reiff M Castille DM Muenzenmaier K Link B Childhood abuse and the content of adult psychotic symptoms. Psychol Trauma (2012) 4:356–69. 10.1037/a0024203

[19] Bhavsar V Boydell J Murray R Power P. Identifying aspects of neighbourhood deprivation associated with increased incidence of schizophrenia. Schizophr Res (2014) 156(1):115–21. 10.1016/j.schres.2014.03.014 24731617

[20] Morgan C Charalambides M Hutchinson G Murray RM. Migration, ethnicity, and psychosis: toward a sociodevelopmental model. Schizophr Bull (2010) 36(4):655–64. 10.1093/schbul/sbq051 20513653 PMC2894585

[21] Bebbington P Wilkins S Jones P Foerster A Murray R Toone B Life events and psychosis. Initial results from the Camberwell Collaborative Psychosis Study. Br J Psychiatry (1993) 162:72–9. 10.1192/bjp.162.1.72 8425143

[22] Dean K Murray RM Environmental risk factors for psychosis. Dialogues Clin Neurosci (2005) 7(1):69–80. 16060597 10.31887/DCNS.2005.7.1/kdean PMC3181718

[23] van Nierop M Lataster T Smeets F Gunther N van Zelst C de Graaf R Psychopathological mechanisms linking childhood traumatic experiences to risk of psychotic symptoms: analysis of a large, representative population-based sample. Schizophr Bull (2014) 40(Suppl 2):S123–30. 10.1093/schbul/sbt150 24562491 PMC3934395

[24] Bell CC Chimata R. Prevalence of neurodevelopmental disorders among low-income African Americans at a clinic on Chicago’s South Side. Psychiatr Serv (2015) 66(5):539–42. 10.1176/appi.ps.201400162 25726976

[25] Morgan C Reininghaus U Fearon P Hutchinson G Morgan K Dazzan P Modelling the interplay between childhood and adult adversity in pathways to psychosis: initial evidence from the AESOP study. Psychol Med (2014) 44(2):407–19. 10.1017/S0033291713000767 23590972 PMC4081841

[26] Muenzenmaier KH Seixas AA Schneeberger AR Castille DM Battaglia J Link BG. Cumulative effects of stressful childhood experiences on delusions and hallucinations. J Trauma Dissociation (2015) 16(4):442–62. 10.1080/15299732.2015.1018475 25895104

[27] McCarthy-Jones S Longden E. Auditory verbal hallucinations in schizophrenia and post-traumatic stress disorder: common phenomenology, common cause, common interventions? Front Psychol (2015) 6:1071. 10.3389/fpsyg.2015.01071 26283997 PMC4517448

[28] Pilton M Varese F Berry K Bucci S. The relationship between dissociation and voices: a systematic literature review and meta-analysis. Clin Psychol Rev (2015) 40:138–55. 10.1016/j.cpr.2015.06.004 26117061

[29] Anglin DM Polanco-Roman L Lui F. Ethnic variation in whether dissociation mediates the relation between traumatic life events and attenuated positive psychotic symptoms. J Trauma Dissociation (2015) 16(1):68–85. 10.1080/15299732.2014.953283 25365538

[30] Berg AO Aas M Larsson S Nerhus M Hauff E Andreassen OA Childhood trauma mediates the association between ethnic minority status and more severe hallucinations in psychotic disorder. Psychol Med (2015) 45(1):133–42. 10.1017/S0033291714001135 25065296

[31] Oh H Cogburn CD Anglin D Lukens E DeVylder J. Major discriminatory events and risk for psychotic experiences among black Americans. Am J Orthopsychiatry (2016) 86(3):277–85. 10.1037/ort0000158 26963179

[32] Metzl JM The Protest Psychosis: How Schizophrenia Became a Black Disease. Boston, MA: Beacon Press (2010).

[33] Myers NA Ziv T “No one ever even asked me that before”: autobiographical power, social defeat, and recovery among African Americans with lived experiences of psychosis. Med Anthropol Q (2016) 30(3):395–413. 10.1111/maq.12288 26990015

[34] Strauss A Corbin J Basics of Qualitative Research. Newbury Park, CA: SAGE (1990). 15 p.

[35] First MB Spitzer RL Gibbon M Williams JBW Structured Clinical Interview for DSM-IV-TR Axis I Disorders, Research Version, Patient Edition (SCID-I/P). New York: Biometrics Research, New York State Psychiatric Institute (2002).

[36] Gray MJ Litz BT Hsu JL Lombardo TW. Psychometric properties of the life events checklist. Assessment (2004) 11(4):330–41. 10.1177/1073191104269954 15486169

[37] Kay SR Fiszbein A Opler L. The positive and negative syndrome scale (PANSS) for schizophrenia. Schizophr Bull (1987) 13(2):261–76. 10.1093/schbul/13.2.261 3616518

[38] Lindenmayer JP Bernstein-Hyman R Grochowski S. Five-factor model of schizophrenia. Initial validation. J Nerv Ment Dis (1994) 182(11):631–8. 10.1097/00005053-199411000-00006 7964671

[39] Lehoux C Gobeil MH Lefebvre AA Maziade M Roy MA The five-factor structure of the PANSS: a critical review of its consistency across studies. Clin Schizophre Relat Psychoses (2009) 3(2):103–10. 10.3371/CSRP.3.2.5

[40] Orfield G Lee C Why Segregation Matters: Poverty and Educational Inequality (The Civil Rights Project ed.). Massachusetts: Harvard University (2005).

[41] Wilson WJ Being Poor, Black, and American the Impact of Political, Economic, and Cultural Forces. American Educator (2011).

[42] Hatch SL Dohrenwend BP. Distribution of traumatic and other stressful life events by race/ethnicity, gender, SES and age: a review of the research. Am J Community Psychol (2007) 40(3–4):313–32. 10.1007/s10464-007-9134-z 17906927

[43] Marrast L Himmelstein DU Woolhandler S. Racial and ethnic disparities in mental health care for children and young adults: a national study. Int J Health Serv (2016) 46(4):810–24. 10.1177/0020731416662736 27520100

[44] Breslau N Davis GC Andreski P Peterson E. Traumatic events and posttraumatic stress disorder in an urban population of young adults. Arch Gen Psychiatry (1991) 48(3):216–22. 10.1001/archpsyc.1991.01810270028003 1996917

[45] Breslau N Kessler RC Chilcoat HD Schultz LR Davis GC Andreski P. Trauma and posttraumatic stress disorder in the community: the 1996 Detroit area survey of trauma. Arch Gen Psychiatry (1998) 55(7):626–32. 10.1001/archpsyc.55.7.626 9672053

[46] McCabe KA Gregory SS Elderly victimization: an examination beyond the FBI’s index crimes. Res Aging (1998) 20:363 10.1177/0164027598203005

[47] Turner RJ Lloyd DA. Stress burden and the lifetime incidence of psychiatric disorder in young adults: racial and ethnic contrasts. Arch Gen Psychiatry (2004) 61(5):481–8. 10.1001/archpsyc.61.5.481 15123493

[48] Alegria M Chatterji P Wells K Cao Z Chen CN Takeuchi D Disparity in depression treatment among racial and ethnic minority populations in the United States. Psychiatr Serv (2008) 59(11):1264–72. 10.1176/appi.ps.59.11.1264 18971402 PMC2668139

[49] Horvitz-Lennon M Frank RG Thompson W Baik SH Alegria M Rosenheck RA Investigation of racial and ethnic disparities in service utilization among homeless adults with severe mental illnesses. Psychiatr Serv (2009) 60(8):1032–8. 10.1176/appi.ps.60.8.1032 19648189 PMC2898183

[50] Metzl JM Herzig RM Medicalisation in the 21st century: introduction. Lancet (2007) 369(9562):697–8. 10.1016/S0140-6736(07)60317-1 17321315

[51] Morrison AP Frame L Larkin W Relationships between trauma and psychosis: a review and integration. Br J Clin Psychol (2003) 42(Pt 4):331–53. 10.1348/014466503322528892 14633411

[52] van Nierop M Viechtbauer W Gunther N van Zelst C de Graaf R Ten Have M Childhood trauma is associated with a specific admixture of affective, anxiety, and psychosis symptoms cutting across traditional diagnostic boundaries. Psychol Med (2015) 45(6):1277–88. 10.1017/S0033291714002372 25273550

[53] Shevlin M Dorahy MJ Adamson G. Trauma and psychosis: an analysis of the National Comorbidity Survey. Am J Psychiatry (2007) 164(1):166–9. 10.1176/ajp.2007.164.1.166 17202562

[54] Fleming MP Martin CR Trauma exposure, schizophrenia symptoms, and the stress vulnerability model. In: Martin CR Preedy VR Patel VB, editors. Comprehensive Guide to Post-Traumatic Stress Disorders. Switzerland: Springer International Publishing (2016). p. 205–29. 10.1007/978-3-319-08359-9_40

[55] Biafora FA Warheit GJ Vega WA Gil AG Stressful life events and changes in substance use among a multiracial/ethnic sample of adolescent boys. J Community Psychol (1994) 22:296 10.1002/1520-6629(199410)22:4<296::AID-JCOP2290220403>3.0.CO;2-T

[56] Franko DL Striegel-Moore RH Brown KM Barton BA McMahon RP Schreiber GB Expanding our understanding of the relationship between negative life events and depressive symptoms in black and white adolescent girls. Psychol Med (2004) 34(7):1319–30. 10.1017/S0033291704003186 15697058

[57] Longden E Sampson M Read J Childhood adversity and psychosis: generalised or specific effects? Epidemiol Psychiatr Sci (2016) 25(4):349–59. 10.1017/S204579601500044X 26156083 PMC7137611

[58] Shevlin M Houston JE Dorahy MJ Adamson G. Cumulative traumas and psychosis: an analysis of the national comorbidity survey and the British Psychiatric Morbidity Survey. Schizophr Bull (2008) 34(1):193–9. 10.1093/schbul/sbm069 17586579 PMC2632373

[59] Frissen A Lieverse R Drukker M van Winkel R Delespaul P GROUP Investigators. Childhood trauma and childhood urbanicity in relation to psychotic disorder. Soc Psychiatry Psychiatr Epidemiol (2015) 50(10):1481–8. 10.1007/s00127-015-1049-7 25895686 PMC4589545

[60] Catone G Pisano S Broome M Lindau JF Pascotto A Gritti A Continuity between stressful experiences and delusion content in adolescents with psychotic disorders – a pilot study. Scand J Child Adolesc Psychiatry Psychol (2016) 4(1):14–22. 10.21307/sjcapp-2016-004

[61] Ellett L Freeman D Garety PA. The psychological effect of an urban environment on individuals with persecutory delusions: the Camberwell walk study. Schizophr Res (2008) 99(1–3):77–84. 10.1016/j.schres.2007.10.027 18061407

[62] Wickham S Taylor P Shevlin M Bentall RP. The impact of social deprivation on paranoia, hallucinations, mania and depression: the role of discrimination social support, stress and trust. PLoS One (2014) 9(8):e105140. 10.1371/journal.pone.0105140 25162703 PMC4146475

[63] Misiak B Moustafa AA Kiejna A Frydecka D. Childhood traumatic events and types of auditory verbal hallucinations in first-episode schizophrenia patients. Compr Psychiatry (2016) 66:17–22. 10.1016/j.comppsych.2015.12.003 26995231

[64] Morgan C Gayer-Anderson C. Childhood adversities and psychosis: evidence, challenges, implications. World Psychiatry (2016) 15(2):93–102. 10.1002/wps.20330 27265690 PMC4911761

[65] Jones N Kelly T Shattell M. God in the brain: experiencing psychosis in the postsecular United States. Transcult Psychiatry (2016) 53(4):488–505. 10.1177/1363461516660902 27495312

[66] Chen YY Koenig H Traumatic stress and religion: is there a relationship? A review of empirical findings. J Relig Health (2006) 45(3):371–81. 10.1007/s10943-006-9040-y

[67] Hood RW Jr Hill PC Spilka B The Psychology of Religion: An Empirical Approach. New York, NY: Guilford Press (2009).

[68] Park CL Handbook of the Psychology of Religion and Spirituality. Guilford Press (2005).

[69] Pargament KI Lomax JW Understanding and addressing religion among people with mental illness. World Psychiatry (2013) 12(1):26–32. 10.1002/wps.20005 23471791 PMC3619169

[70] Pargament K The Psychology of Religion and Coping: Theory, Research, and Practice. New York: Guilford Press (1997).

[71] Tepper L Rogers SA Coleman EM Malony HN. The prevalence of religious coping among persons with persistent mental illness. Psychiatr Serv (2001) 52(5):660–5. 10.1176/appi.ps.52.5.660 11331802

[72] Fallot RD. Spirituality and religion in recovery: some current issues. Psychiatr Rehabil J (2007) 30(4):261–70. 10.2975/30.4.2007.261.270 17458450

[73] Read J Agar K Argyle N Aderhold V. Sexual and physical abuse during childhood and adulthood as predictors of hallucinations, delusions and thought disorder. Psychol Psychother (2003) 76(Pt 1):1–22. 10.1348/14760830260569210 12689431

[74] Freeman D Fowler D. Routes to psychotic symptoms: trauma, anxiety and psychosis-like experiences. Psychiatry Res (2009) 169(2):107–12. 10.1016/j.psychres.2008.07.009 19700201 PMC2748122

[75] Read J Argyle N. Hallucinations, delusions and thought disorder among adult psychiatric inpatients with a history of child abuse. Psychiatr Serv (1999) 50:1467–72. 10.1176/ps.50.11.1467 10543857

[76] Upthegrove R Broome MR Caldwell K Ives J Oyebode F Wood SJ Understanding auditory verbal hallucinations: a systematic review of current evidence. Acta Psychiatr Scand (2015) 133:352–67. 10.1111/acps.12531 26661730

[77] Longden E Read J Social adversity in the etiology of psychosis: a review of the evidence. Am J Psychother (2016) 70(1):5–33. 27052604 10.1176/appi.psychotherapy.2016.70.1.5

[78] Lu W Yanos PT Silverstein SM Mueser KT Rosenberg SD Gottlieb JD Public mental health clients with severe mental illness and probable posttraumatic stress disorder: trauma exposure and correlates of symptom severity. J Trauma Stress (2013) 26(2):266–73. 10.1002/jts.21791 23508645 PMC3888861

[79] Darves-Bornoz JM Lemperiere T Degiovanni A Gaillard P. Sexual victimization in women with schizophrenia and bipolar disorder. Soc Psychiatry Psychiatr Epidemiol (1995) 30(2):78–84. 10.1007/BF00794947 7754420

[80] Fisher HL Craig TK Fearon P Morgan K Dazzan P Lappin J Reliability and comparability of psychosis patients’ retrospective reports of childhood abuse. Schizophr Bull (2011) 37(3):546–53. 10.1093/schbul/sbp103 19776204 PMC3080697

[81] Goodman LA Thompson KM Weinfurt K Corl S Acker P Mueser KT Reliability of reports of violent victimization and posttraumatic stress disorder among men and women with serious mental illness. J Trauma Stress (1999) 12(4):587–99. 10.1023/A:1024708916143 10646178

